# Supplementary material for: Virulent Phages Isolated from a Smear-Ripened Cheese Are Also Detected in Reservoirs of the Cheese Factory
Source: Viruses. 2022 Jul 25;14(8):1620. doi: 10.3390/v14081620 (PMC9331655; doi:10.3390/v14081620)
Supplement: Supplementary file 1 [file viruses-14-01620-s001.zip › Supplementary Tables.pdf]

Table S1. Sensitive/resistant strains to Voltaire and Montesquieu

Strain or isolate	Source	Sensitivity to Voltaire	Sensitivity to Montesquieu
16	Studied cheese	-	-
26	Studied cheese	-	+
33	Studied cheese	-	+
43	Studied cheese	-	-
51	Studied cheese	-	+
52	Studied cheese	-	+
53	Studied cheese	-	+
65	Studied cheese	+	-
119	Studied cheese	+	-
135	Studied cheese	-	-
183	Studied cheese	-	-
186	Studied cheese	-	+
201	Studied cheese	-	+
G. arilaitensis Re117 (DSM 16368)	Reblochon cheese	-	-
G. bergerei Ca106 (DSM 16367)	Camembert cheese	-	-
G. nicotianae DSM 20123	Air of tobacco warehouses	-	-
G. uratoxydans DSM 20647	Humus soil	-	-

Table S2. AMGs encoded by each phage

Phage	Number of CDS	CDS	Function
Voltaire	0	/	/
Montesquieu	2	MONT_40	ABC (ATP-Binding Cassette) transporter
		MONT_41	ACCD (Aminocyclopropane-1-carboxylate deaminase)
Rousseau	3	ROUS_25	Putative glutaminyl cyclase
		ROUS_48	Thioredoxin-like protein
		ROUS_51	S-adenosyl-dependent methyltransferase
Diderot	1	DID_9	Ribonuclease Z
D'Alembert	7	DAL_34	Thioredoxin
		DAL_47	Putative antitoxin
		DAL_102	Transglycosylase
		DAL_103	Chaperonin
		DAL_106	S-adenosyl-dependent methyltransferase
		DAL_114	Ntn_hydrolase-like protein
		DAL_134	Nucleoside Triphosphate Pyrophosphohydrolase
