# Supplementary figures and images for: Virulent Phages Isolated from a Smear-Ripened Cheese Are Also Detected in Reservoirs of the Cheese Factory
Source: Viruses. 2022 Jul 25;14(8):1620. doi: 10.3390/v14081620 (PMC9331655; doi:10.3390/v14081620)

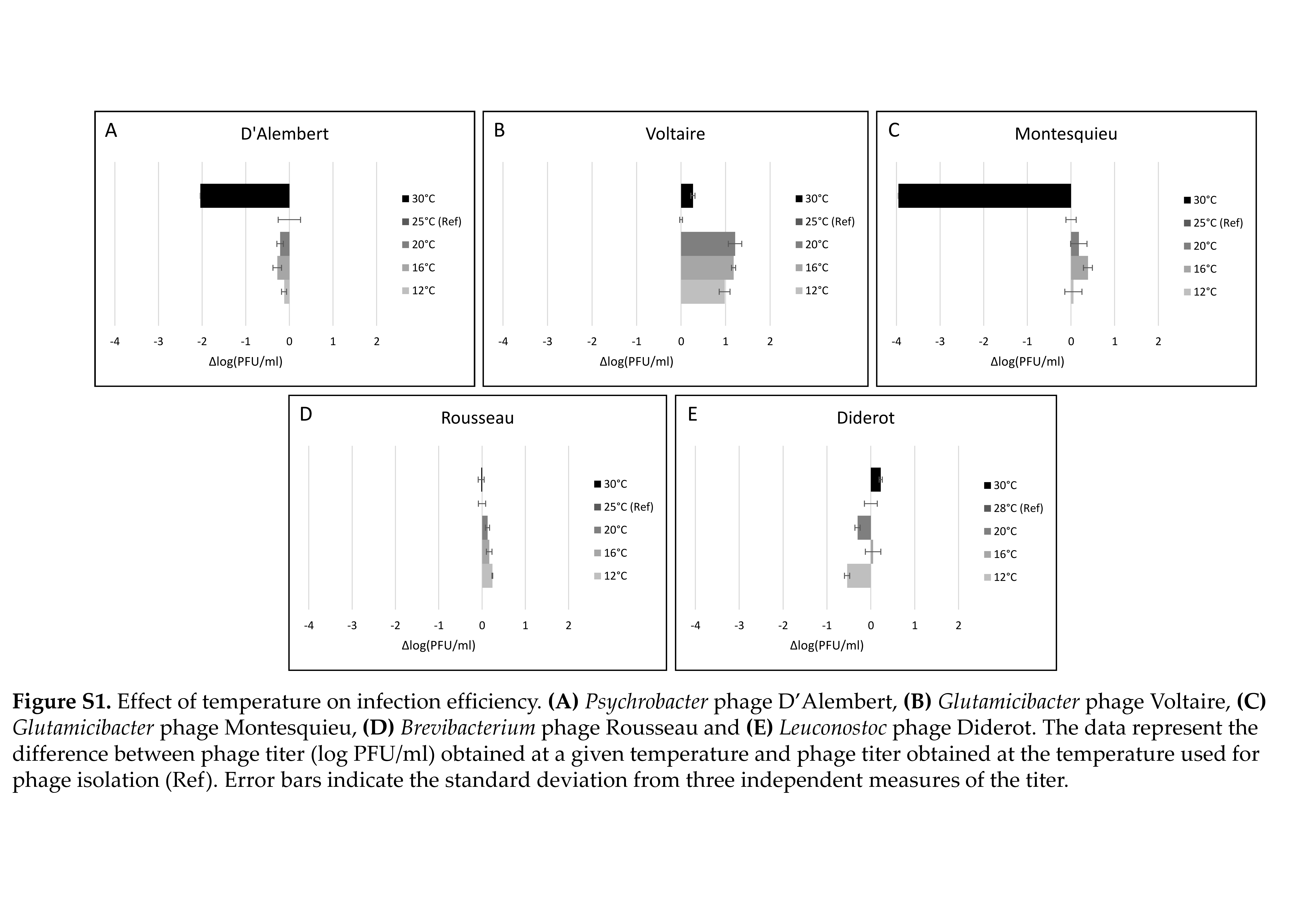

Supplement: Supplementary file 1 [file viruses-14-01620-s001.zip › Figure S1.tif]
